# Domain control of carrier density at a semiconductor-ferroelectric interface

**DOI:** 10.1038/srep14740

**Published:** 2015-10-19

**Authors:** I. B. Misirlioglu, M. Yildiz, K. Sendur

**Affiliations:** 1 Faculty of Engineering and Natural Sciences, Sabancı University, Tuzla/Orhanli, İstanbul 34956 Turkey

## Abstract

Control of charge carrier distribution in a gated channel via a dielectric layer is currently the state of the art in the design of integrated circuits such as field effect transistors. Replacing linear dielectrics with ferroelectrics would ultimately lead to more energy efficient devices as well as the added advantage of the memory function of the gate. Here, we report that the channel-off/channel-on states in a metal/ferroelectric/semiconductor stack are actually transitions from a multi domain state to a single domain state of the ferroelectric under bias. In our approach, there is no a priori assumption on the single or multi-domain nature of the ferroelectric layer that is often neglected in works discussing the ferroelectric-gate effect on channel conductivity interfacing a ferroelectric. We also predict that semiconductor/ferroelectric/semiconductor stacks can function at even lower gate voltages than metal/ferroelectric/semiconductor stacks when an n-type semiconductor is placed between the ferroelectric and the gate metal. Our results suggest the ultimate stability of the multidomain state whenever it interfaces a semiconductor electrode and that a switchable single domain state may not be necessary to achieve effective control of conductivity in a p-type channel. Finally, we discuss some experimental results in the literature in light of our findings.

Gated channels in semiconductor junctions where the passage of current upon a gate bias normal to the channel plane occurs remains the basis for logic devices for information processing and data storage such as the field effect transistor (FET) and solid state memories. Following developments in thin film processing and litography methods that allowed deposition and patterning of high quality film structures on substrates, ferroelectric (FE) materials created great interest among research groups working on semiconductor devices, including efforts to incorporate them into random access memories, photovoltaics, rectifiers, graphene based devices, tunnel junctions and FETs^1^,^2^,^3^,^4^,^5^,^6^,^7^,^8^,^9^,^10^,^11^,^12^,^13^,^14^,^15^,^16^,^17^,^18^,^19^,^20^,^21^,^22^. The driving force behind this motivation was that ferroelectrics have the highest dielectric constant of any known crystalline material that could comprise a highly efficient gate, a polarization dependent current-valve function and the capability to retain a charge owing to their remnant polarization under zero bias. Very recently, Ponath *et al.* has shown experimentally that a ferroelectric layer can alter the carrier distribution in a Ge substrate near the FE/Ge interface, demonstrating the feasibility of FEs for FET channel control^23^. Moreover, FEs have also entered the agenda of groups developing reliable solid state memories owing to the low voltage operation in a FE layer compared to current technologies such as NAND or NOR flash memory^17^,^24^,^25^. This approach, however, brought about the unsubstantiated conjecture that the unidirectional polarization normal to the FE/semiconductor (FE/SC) interface would be easy to tailor by external bias via an electrode with respect to the semiconductor base or substrate. Several other parameters come into play, however, when a ferroelectric is deposited as a film on a misfitting substrate, such as strain effect, thickness effect, and screening length of the electrode(s), that depend on the doping of the semiconductor base, dielectric constant and work function of the electrodes. As a result, the ferroelectric can behave in a manner inconsistent with its bulk characteristics, such as Curie temperature. The stabilization of a multidomain (MD) state in FE thin films has been predicted due to the finite screening of the electrodes in the early 1980s and conserved its validity until now followed by further theoretical analysis^26^,^27^,^28^,^29^ and recent experimental observations^30^. The presence of ionized point defects such as vacancies and impurities in the FE layer, however, can alter the tendencies put forth by the authors studying FE films in the fully insulating dielectric limit^31^. For instance, changes in the characteristics of domain structures as well as the formation of depletion zones and Ohmic-like interfaces from the FE side have been frequently reported by experimentalists followed by detailed theoretical analyses both via atomistic^32^ and continuum limit methods^33^ where the two approaches agree very well in explaining the spatial dependency of polarization and carrier distribution despite the former being a microscopic theory.

It is important to carefully consider the single domain—multi domain (SD-MD) stability criteria while incorporating FEs into semiconductor devices such as FETs, especially given that the use of thin insulating layers at the channel-ferroelectric interface appears to be a common practice to prevent leakage during high bias to switch the FE polarization for gate control (Please see refs 22,23,34). The presence of an insulating barrier layer between the FE and the channel would also trigger electrical domains to minimize depolarizing fields emanating from bad screening of polarization charges at the insulator/FE interface. The geometry and anisotropy of the ferroelectric polarization dictates the pattern of the domain structure to minimize the internal electrostatic fields emanating from the partial screening of polarization charges at coordinates where the polarization vector is normal to the termination plane of the material, such as the FE/SC interface. The idea that an MD state might not exist in relatively thick structures whereby thickness defined here is parallel to the axis of the ferroelectric polarization does not immediately appeal to researchers as the dimensions of the devices into which ferroelectrics are to be integrated are constantly shrinking. Moreover, it is not immediately apparent with thick structures as competition exists between electrostatic energy, domain wall energy and the possible deviation of FE polarization from single domain values within the domains, often requiring extensive numerical analysis before qualitative estimates. A good example of the latter statement is the ferroelectric phase stability in ferroelectric-paraelectric (FE-PE) superlattices wherein it was shown that only thin layers sustain a SD state and this depends on the electrodes, dielectric constant of the PE layer and domain wall energy^35^,^36^,^37^. This may be positive news for groups working on device design as thin FE layers in SD state will exhibit a single sign of polarization, however the temperature range within which this state is stable (or even metastable) needs to be identified. Recent results found that a negative strain (compressive) in a ferroelectric film below the critical thickness of dislocation formation (usually at the order of a few nanometers) can render a SD state stable but this requires interfacing at least on one side a high-*k* layer such as SrTiO_3_ or Ba,Sr,TiO_3_ solid solutions. Top-bottom interface asymmetry could also be exploited to generate a built-in bias in very thin FE layers to enforce an imprinted SD state such as in FE tunnel junctions^10^,^11^. Whether the polarization constituting this SD state will be of ferroelectric origin needs to be understood as Liu *et al.* recently predicted a non-ferroelectric polar phase for very thin tunnel junctions that is likely the built-in polarization that exists above the *T*_*C*_^38^.

In this work, we explore the stability of the MD state of an FE layer when interfacing a semiconductor and place emphasis on carrier distribution and band bending in metal/FE/SC (m/FE/SC) and SC/FE/SC stacks using a continuum media method. The SC considered in the former is a low-to-moderately doped p-type one (pSC) with conduction and valence band density of states and a bandgap similar to that of Si. The n-type SC (nSC), when acting as the bottom electrode, is heavily doped at all times (10^20^ cm^−3^) and has the same band parameters as Si. The metal contact to the nSC and pSC is an ideal metal with an unphysical thickness of a dead layer (≪1 Å) meaning that the electric fields cannot penetrate inside. The picture in both types of systems is quite different regarding the stability of an SD state. The MD state appears to be stable both in the m/FE/pSC and nSC/FE/pSC stacks under zero bias except that the latter has a different space charge distribution due to the asymmetry of the electrodes. The MD state of the FE layer in both stacks at zero bias does not allow conduction in the channel due to discontinuities of the electron density at the FE/pSC interface where the domains terminate. A critical bias, however, exists that forces the SD state in the FE of the m/FE/pSC stacks, allowing the formation of a local and continous charge density at the FE/pSC interface consisting of electrons in the conducton band, making the channel become conductive. The nSC/FE/pSC stack, on the other hand, remains in an MD state at such bias values but can still induce a continuous sheet of carriers at the FE/pSC interface. An important outcome of our work regarding the latter case is that one may not need to induce an SD state and conduction might start before this bias value when an MD state still exists. In the “Results and Discussion” section, we compare our results to previously published experimental data on similar FE/pSC stacks qualitatively and argue that channel control in a pSC via an FE gate using bias values smaller than the coercive bias implies that the domain wall motion is responsible for the reported ranges of “channel-on” and “channel-off” conductivities in previous experimental works. Our results suggest that logic devices such as FETs are directly benefiting from the domains as the off-state in a FE/pSC stack. Moreover, particularly in the case of compensated SCs, a sequence of “hole rich” and “electron rich” regions at the MD-FE/SC interface could be perceived as the generation of nano-sized p-n junctions.

## Theory and Method

**Figure 1:**
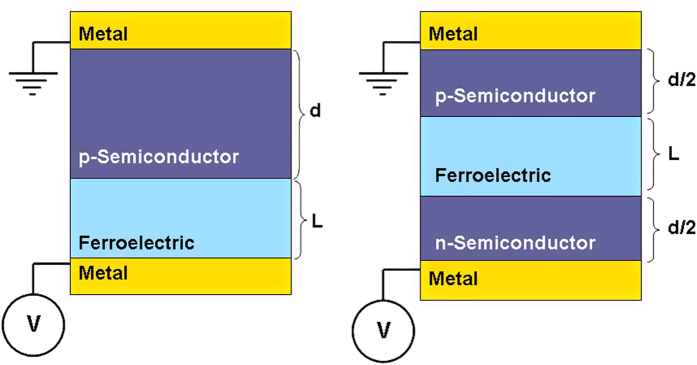
Schematics of the two thin film stacks studied in this work (a) m/FE/pSC and (b) nSC/FE/pSC, both systems with ideal electrodes.

The schematic of the 2 stacks considered in our work is provided in Fig. 1. *L* is the thickness of the FE layer, and *d* is the total thickness of the SC layers. We focus our attention on the m/FE/pSC and nSC/FE/pSC stacks. The top and bottom boundaries are short circuited when under zero bias or the top electrode/pSC are connected to a power supply to apply bias. The SC is a p-type with shallow acceptor dopants varying from 10^17^ cm^−3^ to 10^18^ cm^−3^ with each acceptor acquiring one electron from the valence band and FE considered to be intrinsic with no ionizable impurities. The n-type bottom layer, when present, has a 10^20^ cm^−3^ donor dopant concentration and has the same band parameters as the p-type except that the occupancy of the states are determined by dopant types and ionization energies. The semiconducting parameters of the pSC, nSC and the FE are in Table 1. The Maxwell equation in both the FE and SCs is satisfied where


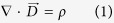


and we can write 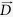, the dielectric displacement vector, as


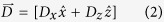


where


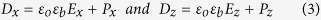


in the FE with *x* and *z* denoting the in-plane and out-of-plane components respectively and,


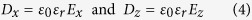


in the pSC and nSC both have a dielectric constant of *ε*_*r*_. In Eqs 2 and 3, *ε*_*o*_ is the permittivity of the vacuum and *ε*_*b*_ is the background dielectric constant (7 in this work^39^,^40^) of the ferroelectric, *E*_*x*_ and *E*_*z*_ are respectively the *x–* and *z–* components of the electric field vector 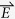 whose components along *x* and *z* are determined from 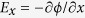 and 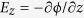, *P*_*x*_ and *P*_*z*_ are the FE polarization components along *x*- and *z*-axes respectively. *ρ* is the total charge density and consists of electrons, holes in both layers and ionized acceptors that are present only in the pSC and ionized donors only in the nSC:


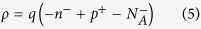


where


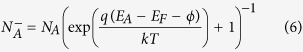



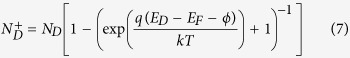



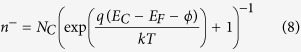



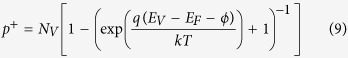



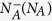
 is the ionized (total) acceptor density in the pSC, 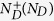 is the ionized (total) donor density in the nSC, *n*^−^ is the electron density, *p*^+^ is the hole density (equal to *not finding* an electron at the max. valence band energy with reference to the Fermi level), *N*_*C*_ is the effective density of states at the bottom of the conduction band, *N*_*V*_ is the effective density of states at the top of the valence band, *E*_*C*_ is the energy of an electron at the bottom of the conduction band, *E*_*V*_ is the energy of an electron at the top of the valence band, *E*_*F*_ is the Fermi level, *ϕ* is the local electrostatic potential and *E*_*A*_ is the ionization energy of the acceptor atom that is taken with respect to the top of the valence band. To carry out the calculations including the band bending in the FE layer, one needs to know *E*_*F*_ of the pSC/FE/pSC stack which can be calculated from the charge neutrality condition, i.e.,


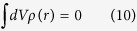


Electrostatic (for the potential) and non-electrostatic boundary conditions (for the polarization) are needed to obtain solutions to the above equations. The boundary conditions for the electrostatic potential is:


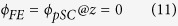


implying the continuity of the potential at the FE/pSC interface and


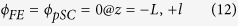


where we assumed all of the charges at the m/FE and m/pSC as well as the m/nSC are compensated and that the metal is ideal. When a bias is applied to the system, potential in the nSC is raised or lowered depending on the sign of bias with respect to the other end of the stack, i. e., the bottom electrode. In both the m/FE/pSC and nSC/FE/pSC stacks, the electrode contacting the pSC is at zero bias (grounded). Before considering any external bias effects, the condition of equality of the electrochemical potential found from Eq. 10 is the same in the FE, and the pSC automatically imposes the condition of the presence of a built-in field and depletion charge distribution as a consequence. Periodic boundary conditions (BCs) are employed along the plane of the structures for both the electrostatic and polarization meaning


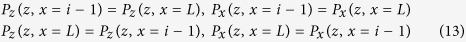


where we have used the first and last “column” of nodes as dummy nodes to connect the *i*’th node to the *L*’th one along the *x*-axis with the exact procedure also carried out for the electrostatic potential along the plane. The polarization boundary conditions at the m/FE, FE/pSC and nSC/FE/pSC interfaces are very important as previously discussed^33^,^41^ and can be expressed as


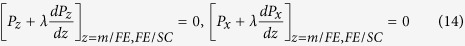


with *z* indicating the coordinates for m/FE and FE/SC interfaces, *λ* is the extrapolation length determining the extent of the change of polarization along the film normal at the interface and is a parameter implying how strongly ferroelectricity is suppressed near the interfaces (taken as 3 nm here based on previous reports^42^). While the electric field is connected to the dielectric properties of the pSC and nSC and FE via Eq. 1, Landau-Ginzburg Eqs. of state for polarization also have to be satisfied in the FE layer:


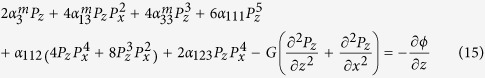



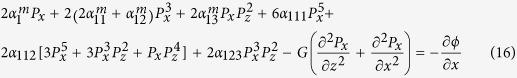


subject to the BCs in Eq. 14. Here 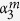, 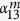, 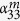, 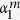, 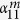, 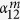 are the renormalized phenomenological thermodynamic coefficients in SI units with 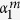 and 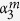 being 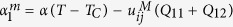 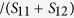 and 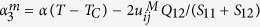 due to renormalization with misfit strain where 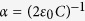, 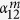 and 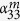 contain the clamping effect of the film, while 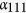, 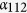, 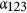 are the dielectric stiffness coefficients in the bulk and can be found for various compositions of Pb,Zr,TiO_3_ in^43^, 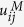 is the misfit strain tensor for a cubic structure taken here as −1% that keeps the FE polarization along the normal of the stack plane. *G* is the gradient energy coefficient and is assumed to be isotropic for convenience, with a value of 6 × 10^−10^ Joule m^3^ C^−2^ 28. The relative dielectric response of the FE layers in the stacks were computed via 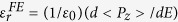
with <>denoting grid average, which can be written in discrete form as:


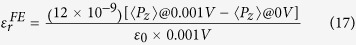


where 1 m*V* is the small signal bias and therefore 1 m*V*/12 × 10^−9^ m is the small signal electric field for a 12 nm thick film in units of m*V*/m. We employ a finite difference discretization in 2D and carry out a Gauss-Seidel iterative scheme to solve the coupled Eqs 1, 4, 5, 15 and 16 simultaneously subject to BCs in Eqs 11. The computation grid consists of n*h* × 200 points where *h* is the distance between the nearest nodes both along *x*- and *z*-axes with a value equal to the cubic state of the FE (~4 angstrom), where n is the number of nodes. In this work n = 30 for the ferroelectric, yielding a FE layer thickness of 12 nm. We terminate the solution after 3000 iterations that yield a difference of about 10^−3^ between two consecutive steps. To ensure the correctness of the numerical solutions, we applied our approach to test cases such as homogeneous FE (λ → ∞) with ideal electrodes and FE sandwiched between 2 PE layers as in refs 35 and 37 where we obtained excellent agreement with our analytical solutions.

## Results and Discussion

### Effect of top-bottom contact asymmetry on the carrier concentration at the FE/pSC interface

**Figure 2:**
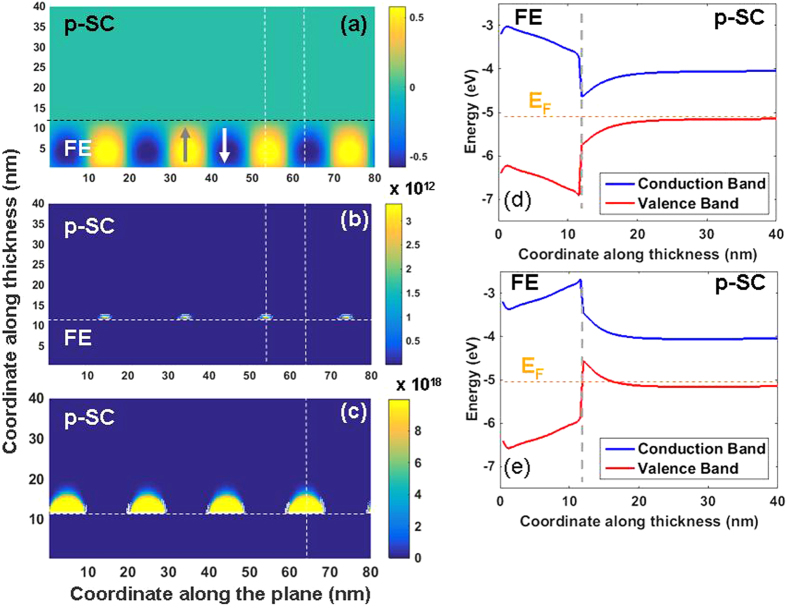
(**a**) Polarization map, (**b**) free electron density, (**c**) hole density, (**d**) flat band structure showing the band bending along the thickness in a −sign domain and (**e**) along a + sign domain for the m/FE/pSC stack with 10^17^ cm^−3^ acceptor density in the pSC at zero bias. Note that in (**b**) free electrons are highly confined. The dashed horizontal line in (**d**,**e**) indicate the Fermi level of the stack (E_F_) computed from Eqs 5.

**Figure 3:**
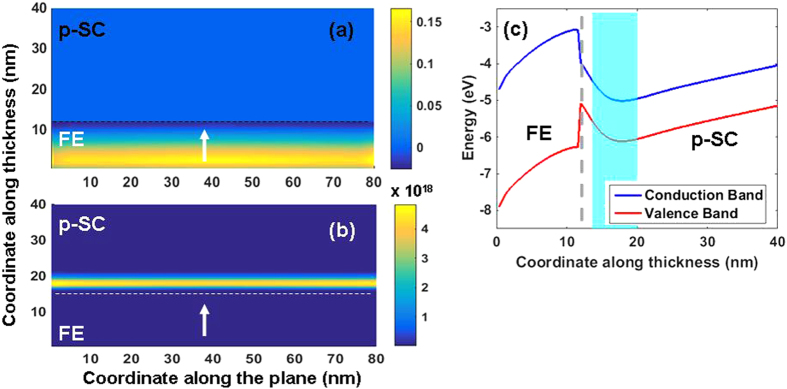
(**a**) Polarization map, (**b**) free electron density, (**c**) flat band structure showing the band bending along the thickness in a +sign domain for the m/FE/pSC stack with 10^17^ cm^−3^ acceptor density in the pSC at 1.5 V bottom electrode bias. The shaded region in (**c**) indicates the electron accumulation at the FE/pSC interface.

A nSC/FE/pSC and m/FE/pSC stack will result in different BCs on the FE layer that can be expected to impact the polarization states and the phase transition temperature of the FE layer. Under zero applied bias, a m/FE/pSC stack will experience a potential drop from one interface to the other depending on the work function difference between the FE and the pSC (under the assumption that the m/FE interface is ideal). As the nSC will have carrier densities much less than a metal in the ideal limit, one might expect a rather strong penetration of the electric field in the former with respect to the metal and, therefore insufficient screening of polarization charges, leading to the MD state. We first provide our results for the domain structure and carrier distributions of the stacks considered in Fig. 1. The m/FE/pSCstack is in MD state at zero bias (bias is on the metal side, pSC is grounded) because of the partial screening of the polarization charge at the FE/pSC interface as mentioned above. The polarization and charge density distribution is given in Fig. 2 where we map the electron and hole density as well as the flat band energy diagrams along the mid-section of a domain after finding the electrostatic potential in the stack whose details are provided in the previous section. Periodic and discrete electron rich/hole rich regions form depending on the sign of the polarization at a given coordinate at the interface as displayed in Fig. 2b,c. The electron rich regions in the pSC are confined to small discrete volumes as they are minority carriers. Band-bending alternates between domains due to the changing sign of the electrostatic potential along *x* as provided in Fig. 2d,e. The alternating sign of potential due to domains creates a formation similar to p-n junctions where electron-rich and hole-rich regions exist. In fact, this should be expected as the polarization termination at the FE/pSC will be screened by conduction electrons and holes in a background of ionized acceptors that are fixed in the pSC. Due to the fact that pSC will always have finite screening of polarization charges, MD formation is inevitable theoretically even under high top-bottom contact asymmetry. Note that here we discuss doped SCs, but not to the extent that they are so heavily doped ultimately leading to metallic behavior, although ideal behavior will still not take place in the latter. The densities of acceptor dopants in the pSC considered here allow free electrons to accumulate near the pSC/FE interface at bias values such as 1.5 V and form a continuous carrier sheet (Fig. 3) as a result of the enforced SD state of the FE in the metal/FE/pSC stack. Removal of the bias puts the FE back in the FE state accompanied by the disapearance of the continuous electron sheet.

**Figure 4:**
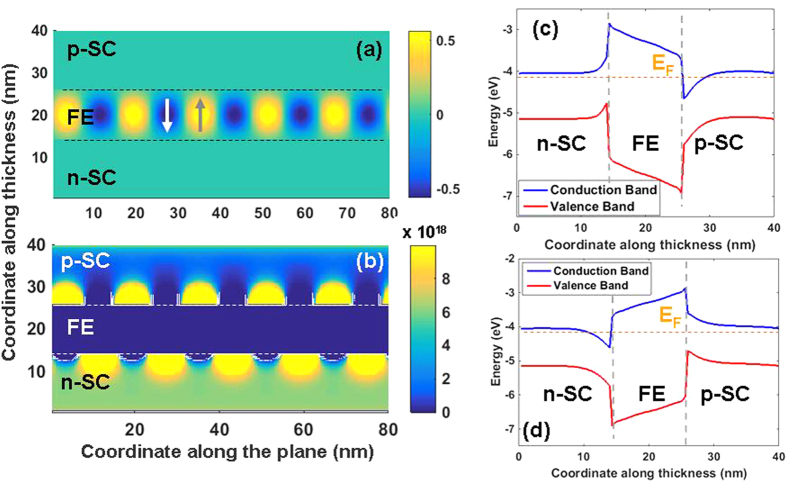
(**a**) Polarization map, (**b**) free electron density, (**c**) hole density, (**d**) flat band structure showing the band bending along the thickness along a +sign domain and (**e**) a −sign domain for the nSC/FE/pSC stack with 10^20^ cm^−3^ donor density in the nSC and 10^17^ cm^−3^ acceptor density in the pSC at 0 V top electrode bias. The dashed horizontal lines in (**c**,**d**) indicate the Fermi level of the stack (E_F_) computed from Eqs 5.

**Figure 5:**
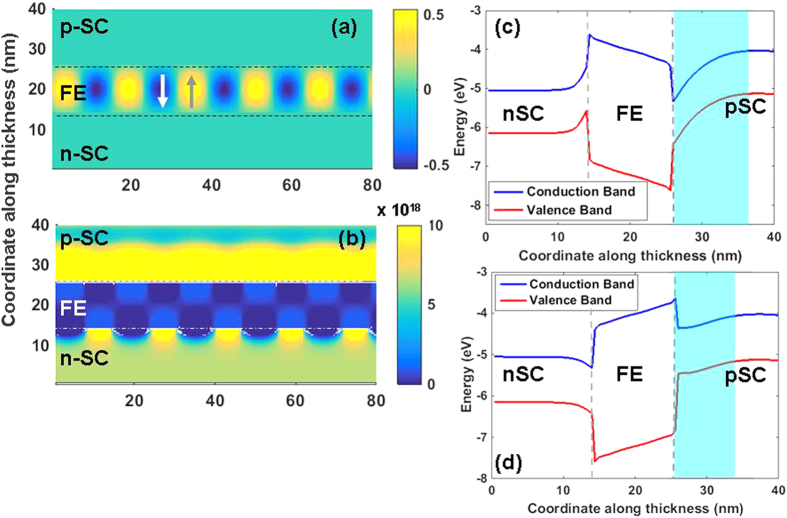
(**a**) Polarization map, (**b**) free electron density, (**c**) flat band structure showing the band bending along the thickness in a +sign domain and (**d**) along a −sign domain for the nSC/FE/pSC stack with 10^20^ cm^−3^ donor density in the nSC and 10^17^ cm^−3^ acceptor density in the pSC at 1 V bottom electrode bias. Note the band bending favoring free electron accumulation at the FE/pSC interface of the −sign domain in the MD state under bias.

Now we focus on the case of nSC/FE/pSC: the behavior of this system heavily depends on the ratio of donors/acceptors in the nSC and pSC respectively. Replacing the ideal metal with an nSC layer never allows a SD state within the bias ranges considered in this work, as we shall show. The polarization, carrier density maps, as well as the flat-band structures are provided in Fig. 4 for the case of 10^20^ cm^−3^ donor and 10^17^ cm^−3^ acceptor density in the nSC and pSC layers, respectively. The bands are assumed to be pinned at the metal interfaces. We noted that a moderate-to-heavily doped nSC on the bottom and a low-to-moderately doped pSC on the top of the FE layer allows precise control of electron density at the FE/pSC interface via a low-to-moderate bias as mentioned above, smaller than the coercive bias for the system. In this stack, we find that a bottom bias on the nSC of around 1 V can lead to a continuous carrier sheet in the pSC, which is more desirable than that of values needed to induce conductivity at the FE/pSC interface via a linear dielectric. The reason for this is that a conventional linear dielectric such as SiO_2_, Al_2_O_3_ or HfO_2_ will have uncomparably small values of “dielectric induction” under the same bias with respect to a FE layer whose dielectric induction has a very strong polarization component. Also note that the domain periods and therefore the domain sequence differ in the nSC/FE/pSC stack from that of the m/FE/pSC one owing to the differences in their Curie temperatures and the extent of top/bottom asymmetry (Compare Figs 2, 3, 4, 5).

A critical bias of around 1 V on the nSC electrode leads to the flat band structure shown in Fig. 5c and electrons roll downhill over the altered electrostatic potential of the conduction band of the pSC towards the pSC/FE interface, generating a conducting region. The holes rise along the valence band edge and accumulate along the flat potential region away from the FE/pSC interface. With the removal of the positive bias on the nSC layer, domain fraction changes and electrons become confined to small discrete volumes without any connectivity along the interface, as shown back in Fig. 4b, thus forming an insulating interface. Our results show that channel conductivity control may be possible in a pSC interfacing a FE and that polarization switching is not necessary in the nSC/FE/SC. Additionally, the channel conductivity we discuss here occurs when the FE layer is in an MD state at all times where a bias only causes slight changes in domain fractions sufficient to form on/off states at the FE/pSC interface.

Horiuchi *et al.* investigated the field effect induced by a ferroelectric layer in FETs^34^. Although theirs is a device-related study, the results presented have implications for our study. In that work, FETs with Pt/SrBiTa_2_O_5_/HfO_2_-Al_2_O_3_ gate stacks on p-type Si substrates were grown and studied. The HfO_2_-Al_2_O_3_ was grown as an insulator between the p-type Si and the FE layer, possibly in order to prevent leakage across the gate. Measurable data retention times of around 30 days were reported in addition to channel-on/channel-off current ratios of the order of 10^6^ (extrapolated to 10^4^ after 10 years) with the emphasis that the authors did not require polarization saturation to control the drain current after 30 days of cycling bias on the stack^34^. Interestingly, they reported that a gate bias of about +2 V was enough to generate significant drain current that is likely below the coercive bias for their films. One must note that the polarization, when in SD-FE state, is weakly dependent on an applied field and despite the exponential dependence of the channel carrier density on the gate bias, on/off drain current ratios of 10^7^–10^8^ are not feasible without switching the total FE polarization, a difficult task with a +2 V gate bias for a 450 nm thick SrBiTa_2_O_5_ with 6–7 nm HfO_2_-Al_2_O_3_ insulating buffer layer that will limit the electric fields achieved in the FE (as a strong potential drop is expected to occur in the insulating HfO_2_-Al_2_O_3_ layer). We argue that this implies a domain driven mechanism that can cause on/off current ratios reported in ref. 33 due to continuous/discontinuous electron density formation at the interface, hence the presence of electrical domains as we also demonstrate in this paper using a PbZr_0.3_Ti_0.7_O_3_/p-Si interface. The fact that Horiuchi *et al.* observe the memory effect in drain current only on the + gate bias values could be interpreted as an indicator that there is not switching but only MD formation with bias decreasing towards zero following saturation. Apart from discussing device related parameters, it is important to understand that the control of p-type channel conductivity with gate voltages below that required for ferroelectric switching could well be a sign that electrical domains are indeed present but not an obstacle to channel conductivity control.

**Figure 6:**
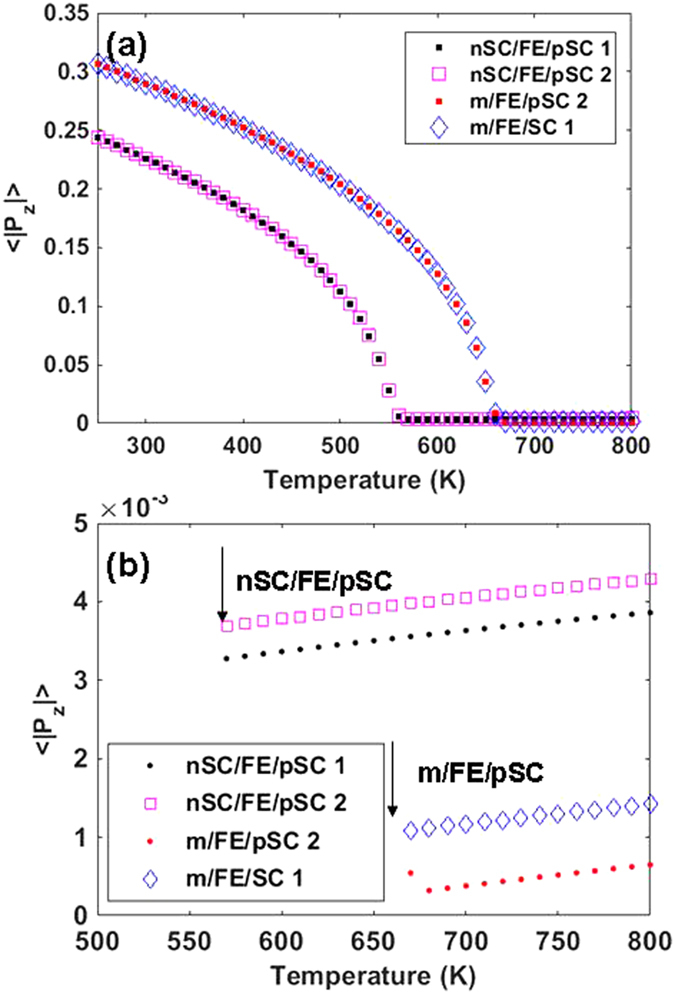
(**a**) 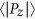 for nSC/FE/pSC and m/FE/pSC stacks with various dopant densities. In the legend, 1 stands for a pSC acceptor density of 10^17^ cm^−3^, 2 stands for pSC acceptor density of 10^18^ cm^−3^. The nSC in the nSC/FE/pSC stack has 10^20^ cm^−3^ donor concentration in all cases. (**b**) displays the non-zero built-in *P*_z_ with a zoom in the high temperature region of the plot in (**a**). Arrows indicate the PE-FE transition temperatures.

During the course of our work, we also noticed a notable mechanism that we extract from our results: there is an interplay between the dopant density in the SC layers and domain wall mobility. Domain wall mobility, to a large extent, is determined by the strength of the depolarizing fields inside the FE layer. As domain wall mobility is important in changing the fraction of + and − domains to control the band bending at the active interfaces, it would appear as if the domain structure is rather hard for low-to-moderate doping of the SC layers due to the partial screening of polarization charges. This is only partially correct because low-to-moderate doping can reduce the PE-FE transition temperature of the FE layer (See Fig. 6) due to insufficient polarization charge screening at the interfaces and this could render the domain pattern relatively “softer” to the applied bias. Moreover, the effect of the presence of ionized dopants in both the nSC and the pSC layers must be considered: in the m/FE/pSC stack, for instance, the holes are pushed far into the pSC when interfacing a −sign domain, exposing the negatively charged but fixed ionized acceptors. A better screening might be expected via such a mechanism if the acceptor concentration is increased, leading to a possibly higher dielectric response as depolarizing fields will be weaker, hence less pinning of domains. We do obtain such a trend as we shall show in the next section but we leave a detailed analysis of this behavior to future work. Furthermore, a strong asymmetry in top-bottom interfaces raises the question of single domain stability in the FE layer, which is also part of future work. Our arguments until here exclude effects of defects and discreteness of the lattice. We next discuss PE-FE transition temperatures and the dielectric response of the FE layer. As the dielectric response depends on domain wall mobility in MD FEs, it is important to study the effects of electrodes and top-bottom asymmetry on domain stability.

### Effect of asymmetry on transition characteristics and dielectric response

Here we give the polarization vs. temperature and dielectric response of the stacks whose schematics were provided in Fig. 1. Two dopant concentrations of the pSC are considered and the nSC dopant concentration, when considered, is fixed at 10^20^ cm^−3^, a relatively high value near the degenerate limit. The lattice average of the absolute value of *P*_z_, namely 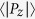, at each site is tracked to compare the transition temperatures since the average polarization with its sign dependency is near zero in all cases in the MD state, making it impossible to detect the transition during cooling runs. From Fig. 6, one can see that the m/FE/pSC stack has a higher *T*_C_ (about 100 degrees higher) and a larger 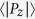 than that for the nSC/FE/pSC stack despite the high dopant concentration in the nSC in the latter. This is because the bottom ideal metal screens the bottom polarization charges well with respect to the nSC contact. Another conclusion to be deduced from Fig. 6a is that the *T*_C_ for the nSC/FE/pSC and m/FE/pSC varies little for the two dopant concentration ratios of the pSC considered in this work. Such a scenario will also depend on the density of states of the SC as much as the dopant concentration where the former puts a limit on how many carriers will contribute to “screening” in theory, and might vary depending on the SC type and structure. Relatively high dopant concentrations in nSC and pSC (such as 10^20^ cm^−3^ in the nSC and 10^18^ cm^−3^ in the pSC) create a built-in field inducing along a built-in *P*_z_ (not of ferroelectric origin) becoming noticable above the *T*_C_ (See Fig. 6b). The magnitude of this field diminishes with lower dopant concentrations in the nSC and pSC as in the case of 10^17^ cm^−3^ in the pSC. Built-in *P*_z_ has a tendency to slightly enhance (See Fig. 6b) as the electron/hole concentration ratio in the nSC and pSC increases, hence the built-in field is slightly stronger with increasing temperature. As we shall see next, the presence of the built-in field for the stacks considered does not favor a particular sign of domain as the small bias signal yields a finite and weak MD response, not a SD response.

**Figure 7:**
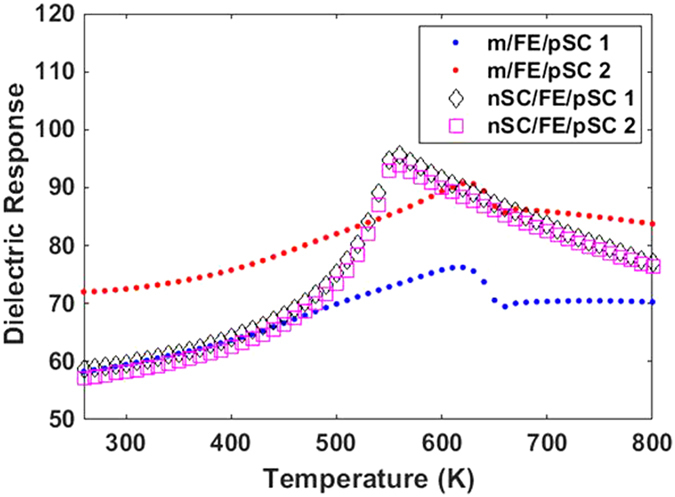
The dielectric response of the FE layer in the m/FE/pSC and nSC/FE/pSC stacks with 1 and 2 denoting 10^17^ cm^−3^, 10^18^ cm^−3^ acceptor density in pSC respectively. The nSC in the nSC/FE/pSC stack has 10^20^ cm^−3^ donor concentration in all cases. The peak points of the dielectric response indicate the PE-FE transition of the relevant stack.

In Fig. 7, we plotted the small signal dielectric response where a bias of 1 mV is applied only to the bottom metal/nSC couple and the top electrode is always at zero bias. Fig. 7 displays the dielectric response of the FE layer in the m/FE/pSC and nSC/FE/pSC stacks. It is clear that we do not obtain the dielectric response values expected for metal/FE/metal capacitors here. Such metal/FE/metal capacitor systems can exhibit a large response in the presence of real electrodes due to the finite screening of polarization charges leading to an MD state and the large dielectric response comes from domain wall motion. Owing to the much larger screening length of the SC layers, the relative dielectric response values we compute barely approach 100 near *T*_C_, meaning that the domain structure is difficult to manipulate via the small signal bias. This tendency can also be understood by looking at the dielectric response of the m/FE/pSC and nSC/FE/pSC stacks: in the latter, we observe the λ-type anomaly in the dielectric response albeit low in magnitude. Such an outcome is interesting because one would expect that the bottom ideal metal electrode in m/FE/pSC eliminates part of the stray fields and some domain wall motion could have been expected. The dielectric response is also smeared strongly in the m/FE/pSC stack due to this top-bottom contact asymmetry, particularly when the dopant concentration in the pSC is increased. An increase in concentration, however, aids the dielectric response in reaching higher values because the higher concentration of ionized acceptors, although fixed in space, contribute to the screening of polarization charges at the interfaces, allowing “less pinning” for domains. There is little change in the *T*_C_ of the m/FE/pSC for an order of magnitude change in acceptor concentration because the PE-FE transition during cooling is into an MD state where the electric fields are confined near the interfaces (not shown here) and the weaker fields inside the FE thus permit a stable polar state.

We note a rather unconventional characteristic in the m/FE/pSC stacks: the λ-type anomaly is replaced by a one that has an unusual temperature dependency especially near *T*_C_ where a step is formed from the PE side of the transition. This type of dielectric response is similar to that of an improper ferroelectric. Improper ferroelectrics are characterized by the ferroelectric polarization appearing as a secondary order parameter for the primary mechanism of the phase transition that could be originating from a symmetry element loss in a crystal that is not directly giving rise to latttice distortions inducing a switchable polar ordering^44^. Despite this, they usually have low spontaneous polarization and can possess a usual dielectric response including near the *T*_C_ region similar to the cases denoted by 1 and 2 of the m/FE/pSC stack in Fig. 7. While it is very likely a coincidence that we predict a dielectric anomaly at *T*_C_ of the m/FE/pSC moderate p-type doping similar in behavior to that of an improper FE crystal, it is worth noting.

It is interesting to see that nSC/FE/pSC structures allow the most efficient channel control with relatively low bottom electrode voltages despite being in the MD state. A low small signal dielectric response from an FE layer in an MD state far below the *T*_C_ would mean domain walls that are hard to move. However, under moderate bias (such as 0.5 V–1 V here), a particular sign of domain can be favored as this bias apparently could overcome or suppress the depolarizing fields to some extent. It is in this regime that a continuous conductive region can be formed at the FE/pSC interface and a small signal dielectric response is not relevant to the formation of this channel in the case of a FE in an MD state. This outcome is in contrast to the case of linear dielectrics gating the channel where a high dielectric constant is desired so that high densities of carriers can accumulate at the channel/gate interface when a gate bias is applied, which is the consequence of Gauss’s law:


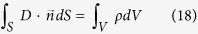


stating that a finite volume, *V*, enclosing the interface of a dielectric and an electrode will contain a total charge equivalent to the component of the dielectric induction along the surface normal 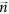 flowing in and out of that volume via the surface, *S*. This fundamental relation must be understood qualitatively to evaluate the outcomes of the current work. As the mobile carriers are mostly available in the SC side, there will be accumulation near the interface. Had there been very low densities of charge, one would then expect strong electric fields to suppress the dielectric induction *D* that would always satisfy Eq. 18. An FE develops a polarization that literally boosts and dominates *D* (See Eq. 3), resulting in what we report in this study, as long as free carriers exist on the electrode (the SC) side. In the event that the electrodes do not provide sufficient density of carriers under zero field, an FE minimizes the strong electric fields that oppose the polarization by splitting into electrical domains. Such a structure might have a low dielectric response if the domain structure is “hard” due to strong depolarizing fields. Therefore, the carrier density at a pSC channel interfacing an FE in an MD state will be a function of the fraction of +sign domains that can only change under moderate bias and not the dielectric response of the FE as long as the FE is far below *T*_C_. The bias at which the continuous electron “sheet” can be formed also depends on the type of electrode interfacing the FE from the opposite side.

## Conclusions

We studied the effect of FE polarization on the carrier distribution in doped pSCs interfacing an FE layer not exceeding 12 nm in thickness. Two different stacks, namely a m/FE/pSC and a nSC/FE/pSC were considered. The electron distribution in the pSC layer in both structures at zero bias is identical in both cases: discrete and small regions of electrons at the termination of the +sign domains of the FE layer at the FE/pSC interface. We showed that the carrier concentration at the FE/pSC interface in the moderately doped nSC/FE/pSC structures can be manipulated more easily with lower bias than in the case of m/FE/pSC stacks despite the former being in an MD state. Being in direct contact with the metal bottom electrode in the m/FE/pSC stack allows an SD state at low-to-moderate bias, not exceeding 1.5 V as the screening of the electric fields at the bottom interface leads to easier domain wall motion. The *T*_C_ of the m/FE/pSC is also higher than that of the nSC/FE/pSC stacks regardless of the dopant concenctrations in the SC layers implying the stronger depolarizing fields in the latter, hence the ultimately stable MD state even under bias. Despite the stronger polarization in the m/FE/pSC stacks and the higher *T*_C_ it exhibits, a bias higher than that required for the nSC/FE/pSC stack is necessary to induce a continuous free electron “sheet” at the FE/pSC interface of the m/FE/pSC for the same dopant concentration of the pSC layers in both types of stacks. This is due to the electrostatic effects in the nSC layer of the nSC/FE/pSC stack and a discrete electron-ionized donor seperation taking effect, leading to the effects we obtained in our work. While we show here that it is possible to benefit from an MD state to induce on/off states in SC layers, likely with much better efficiency and faster than such stacks requiring complete switching of the ferroelectric polarization, more detailed work including atomistic methods are needed to further assess the details of the mechanisms at the FE/SC interfaces. We demonstrate the possibility that the FE layer in an MD state could eliminate the need for “hard to sustain” SD state for prolonged times in “on/off” type device structures. One important factor to consider here is the domain wall mobility under relatively low bias signals: the time duration of a stable, continuous conducting electron sheet at the FE/pSC interface will depend on the “relaxation time” of the domains rather than the “polarization rise time” during the presence of the bias signal on the system. This, in turn should determine the response speeds of such designs employing an FE in an MD state. Given that most current device designs employing an FE to control the channel conductivity rely on total polarization switching, which proceeds with nucleation and growth under a constant bias, we expect tailoring gradual motions of domain walls to be more power efficient. Engineering domains specifically by the choice of SC characteristics the FE is interfacing could stimulate new concepts, eliminating the need for total polarization switching that requires relatively high fields and possibly longer time scales due to the nucleation and growth process. Moreover, we also point out the possibility of creating nano *p-n*-like regions at a ferroelectric/semiconductor interface where carriers are localized due to the band bending caused by the alternating sign of polarization.

## Additional Information

**How to cite this article**: Misirlioglu, I. B. *et al.* Domain control of carrier density at a semiconductor-ferroelectric interface. *Sci. Rep.* **5**, 14740; doi: 10.1038/srep14740 (2015).

## Figures and Tables

**Table 1:** Band parameters, ionization energies of dopants and linear dielectric values the pSC, nSC and the FE used in the calculations.

	E_F_(eV)	N_C_(cm^−3^E^−1^)	N_V_(cm^−3^E^−1^)	N_A_(cm^−3^)	E_A_, E_D_(eV)	E_C_, E_V_(eV)	*ε*_*r*_(*ε*_*b*_ in FE)
pSC	−5.1	10^19^	10^19^	10^17^, 10^18^	−0.05	−4.05, −5.15	10
nSC	−5.1	10^19^	10^19^	10^26^	−0.05	−4.05, −5.15	10
FE	−4.8	10^18^	10^18^	none	none	−3.2, −6.4	7

m^−3^E^−1^ is the unit for density of states (E: Energy unit).
